# Experimental Protocols Used to Mimic Gastrointestinal Protein Digestion: A Systematic Review

**DOI:** 10.3390/nu16152398

**Published:** 2024-07-24

**Authors:** Anna Beatriz Santana Luz, Amanda Fernandes de Medeiros, Gidyenne Christine Bandeira Silva de Medeiros, Grasiela Piuvezam, Thaís Souza Passos, Ana Heloneida de Araújo Morais

**Affiliations:** 1 Biochemistry and Molecular Biology Postgraduate Program, Biosciences Center, Federal University of Rio Grande do Norte, Natal 59064-741, RN, Brazil; annabeatriz@ufrb.edu.br (A.B.S.L.); amanda-nut@hotmail.com (A.F.d.M.); 2 Center for Health Sciences, Federal University of Recôncavo da Bahia, Santo Antônio de Jesus 44430-622, BA, Brazil; 3 Department of Nutrition, Center for Health Sciences, Federal University of Rio Grande do Norte, Natal 59078-970, RN, Brazil; gidyenne.silva@ufrn.br (G.C.B.S.d.M.); thais.passos@ufrn.br (T.S.P.); 4 Public Health Postgraduate Program, Center for Health Sciences, Federal University of Rio Grande do Norte, Natal 59078-970, RN, Brazil; 5 Department of Public Health, Center for Health Sciences, Federal University of Rio Grande do Norte, Natal 59078-970, RN, Brazil; 6 Nutrition Postgraduate Program, Center for Health Sciences, Federal University of Rio Grande do Norte, Natal 59078-970, RN, Brazil

**Keywords:** peptides, in vitro techniques, proteolysis, gastrointestinal tract

## Abstract

Bioactive peptides derived from native proteins modulate physiological processes in the metabolic pathways. Given that multiple protocols in the literature mimic the digestion of dietary components, gathering studies that use such models directed at protein digestion processes is critical. This systematic review aimed to gather evidence that adopted adequate experimental models to simulate human protein digestion. The databases searched were PubMed, Web of Science, ScienceDirect, Embase, Virtual Health Library, and Scopus. A total of 1985 articles were found, resulting in 20 eligible in vitro studies. The Office of Health Assessment and Translation was used to evaluate methodological quality. Seven studies used plant-based protein sources, twelve used animal protein sources, and one used both. The duration of the oral phase varied, although 60% of the studies employed a protein digestion period of 120 min. Amylase, pepsin, and pancreatin enzymes were utilized in 40% of the studies, with pH levels of 7, 3, and 7, respectively, during the oral, gastric, and intestinal phases. The INFOGEST harmonized static model was adopted by 65% of the studies; INFOGEST is the most effective model for simulating gastrointestinal protein processes in humans and can be used to answer several research questions because it describes experimental conditions close to the human physiological situation.

## 1. Introduction

Gastrointestinal digestion is a physical and chemical process in which food is digested by the action of different enzymes, releasing nutrients to be absorbed and used by the body. In the case of proteins, these are hydrolyzed into peptides and amino acids. Thus, knowing how food proteins are digested is essential since it influences their nutritional value and makes their degradation products physiologically relevant [1].

In this context, bioactive peptides are defined as sequences of amino acids contained in a precursor protein whose release allows them to exert biological activities, such as antioxidant, immunomodulator, antidiabetic, and anticancer effects [2]. Thus, such peptides can present different interactions in the human body, modulating physiological processes in various metabolic pathways in the body, for example, inhibiting the action of some enzymes [3]. Therefore, to physiologically understand the body’s reaction to food, one must study the digestion processes in the human digestive tract. These studies can be conducted in humans through aspiration of the stomach or small intestine, which is considered invasive [4,5,6].

According to Santos-Hernández et al. [1], some foods of plant origin, for example, are excellent sources of high-quality protein. In addition, recent review studies presented relevant information about the digestion of proteins of plant origin [7,8]. In this context, systematic reviews are valuable tools for integrating studies on this topic, allowing the evaluation of specificities and differences in treatment procedures to help guide future research [9,10].

Thus, considering that several protocols in the literature mimic the digestion of food constituents, gathering studies that adopt such models aimed at protein digestion processes is extremely important. Therefore, this systematic review aimed to compile experimental protocols that mimic gastrointestinal protein digestion in humans to answer the following question: what digestion models are used to reproduce gastrointestinal protein processes?

## 2. Material and Methods

The construction of this systematic review followed the methodological criteria established by the guidelines described by the Preferred Reporting Items for Systematic Review and Meta-Analysis Protocols (PRISMA-P) [11]. Available online: https://systematicreviewsjournal.biomedcentral.com/articles/10.1186/2046-4053-4-1 (accessed on 24 Jun 2021). The PRISMA-P checklist is available in the File S1. The protocol for constructing the systematic review was registered in the International Prospective Registry of Systematic Reviews, PROSPERO, under CRD42020198709, and published by Luz et al. [12]. The Protocol registered in the PROSPERO is available in the File S2. It is worth mentioning that the registered and published protocol is related to systematic reviews on protein digestion and absorption. However, the present review focuses only on protein digestion.

### 2.1. Search Strategies

Initially, preliminary equations were tested from keywords indexed in Medical Subject Headings (MeSH), aiming to develop high-sensitivity search strategies. The search equations (Table 1) were built with combinations of descriptors related to digestion, peptides or proteins, gastrointestinal tract, animals, or in vitro. Searches were performed on 29 November 2022, using the electronic databases PubMed, Web of Science, Science Direct, Embase, Virtual Health Library, and Scopus.

### 2.2. Eligibility Criteria

Original articles were included according to pre-specified inclusion and exclusion criteria (Table 2).

### 2.3. Data Extraction Process

Articles were imported into the Rayyan application (version 0.1.0) [13], duplicates were excluded, and titles and abstracts were read following the eligibility criteria for this step. Subsequently, the complete texts that passed to the next stage were analyzed, and the studies that met the other inclusion criteria were selected. Two reviewers performed these steps independently, and all included studies were reviewed for data extraction and risk of bias assessment. In the case of discrepancies/conflicts, a third researcher was consulted.

Data extraction was also performed by the same reviewers, independently, in a previously standardized way, building a database using the Microsoft Excel^®^ program version 14.0.0 containing bibliographic information (author and year of publication), type of study, and data related to the protocol adopted to simulate gastrointestinal processes (number of control groups, type of sample used, sample concentration, enzymes used, enzyme concentration, pH, temperature—experimental and interruption, composition and volume of gastrointestinal simulating fluids, and experiment duration).

### 2.4. Bias Risk Assessment and Study Quality Assessment

The studies were evaluated using the OHAT tool, which was adapted [14] and used to assign studies to four categories, namely, definitely low risk of bias (DL), probably low risk of bias (PL), probably high risk of bias (PH, also abbreviated as NR—insufficient information), and definitely high risk of bias (DH) [15]. The tool has eleven questions; however, questions one, two, six, and eleven do not apply because they were not addressed in any of the studies. Therefore, seven questions were used. Two reviewers performed the evaluation independently. In the case of discrepancies/conflicts, a third researcher was consulted. OHAT questions are available in the Table S1.

Thus, with the OHAT, the quality of the studies was evaluated regarding randomization, performance (considering the use of the same vehicle—type, and volume—for the treated and control groups during treatment and blinding), detection (purity and the supplier of the enzymes), attrition (addressing incomplete results such as losses and/or exclusions), reporting (whether all results cited in the methodology and abstract were reported), and other sources of bias (evidence of appropriate statistics and/or adherence to the protocol, comparing the results with the objective and methods mentioned). Finally, if more than four assessments were defined as PH, the study would be excluded due to the high risk of bias [14,15].

Subsequently, aiming to acquire a quantitative assessment based on this tool, numerical values were defined for each of the four evaluations above, which ranged from 01 to 04, where 01—equivalent to no bias (DL), 02—probable low risk of bias (PL), 03—probable high risk of bias, and 04—the presence of bias (DH). Therefore, it was agreed that the higher the OHAT score, the greater the risk of bias in the articles [14,15].

For animal studies, the risk of bias assessment should be performed using the SYRCLE instrument [16]. The researchers were calibrated to carry out the risk of bias assessment. After the evaluation, Cohen’s kappa coefficient of the agreement was applied, which assesses the level of agreement between researchers, obtaining a value of 0.97 [17].

## 3. Results

**Figure 1:**
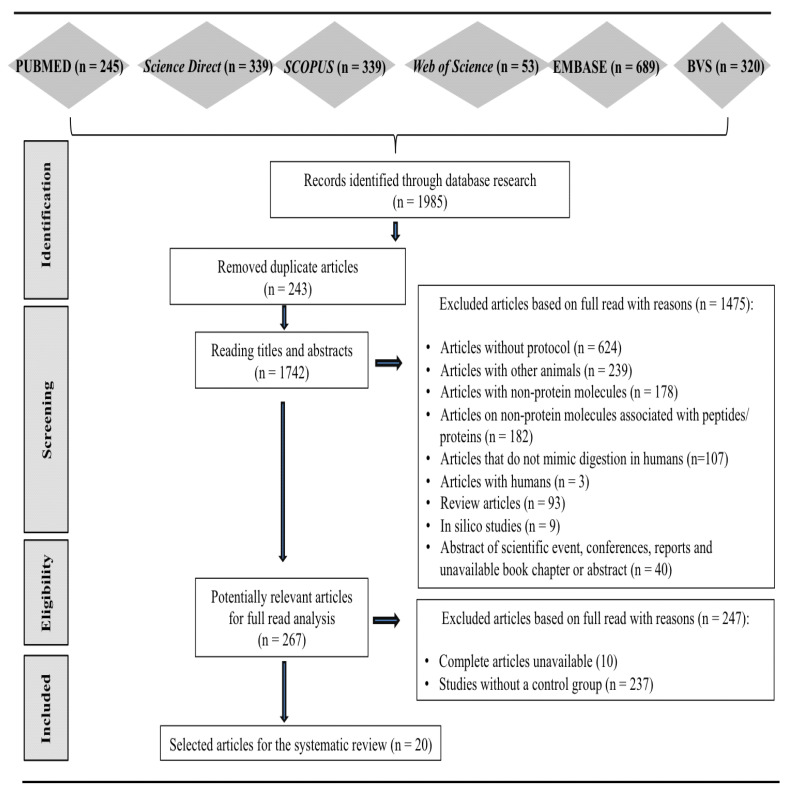
Flowchart of selection steps and study characteristics. Adapted from PRISMA [18]. PRISMA: Preferred Reporting Items for Systematic Reviews and Meta-Analyses.

A total of 1985 articles were identified in the databases, with 243 studies removed because they were duplicates. Of the 1742 articles designated for a reading of the title and abstract, 267 were selected for the entire reading stage. After applying the eligibility criteria, 20 articles were included in the systematic review (Figure 1).

Among the 267 studies selected for full reading, 10 were unavailable for full access. It was checked whether the studies were expanded abstracts published in annals of scientific events, and they were not. Thus, complete articles were requested via Research Gate, but two were not found on the platform. In addition, a search was carried out for profiles registered at Research Gate, and messages were sent directly to more than one author with a record. Finally, emails were sent to the authors of seven articles because it was impossible to find the authors’ emails of three articles published in 1978, 1983, and 1985, which were in Russian. It was also observed that the most recent publication of some of them occurred in 1991 and of another author in 2016. However, these articles were among the documents requested via Research Gate. Until the end of the review, there was no return from any author. Therefore, the ten studies were excluded due to unavailability.

**Figure 2:**
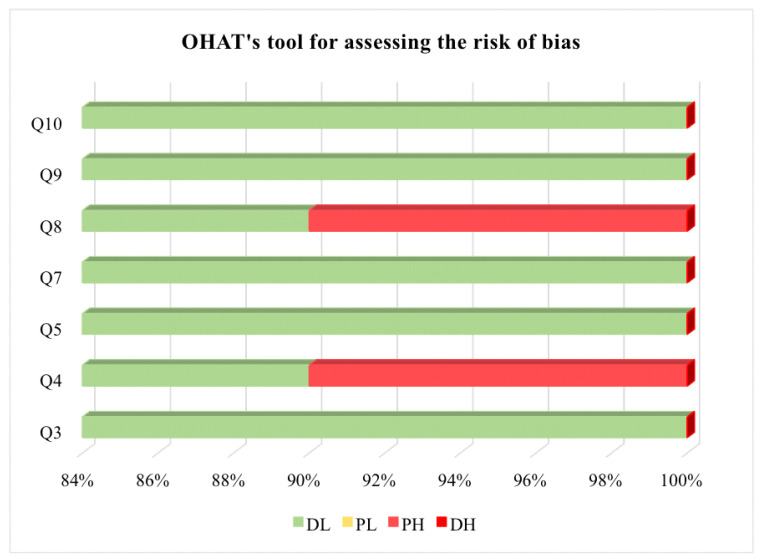
Risk of bias assessment of included studies using the OHAT tool (*n* = 20). Available online: https://ntp.niehs.nih.gov/sites/default/files/ntp/ohat/pubs/riskofbiastool_508.pdf (accessed on 13 December 2022). OHAT: Office of Health Assessment and Translation. DL: definitely low risk of bias; PL: probably low risk of bias; PH: probably high risk of bias; and DH: definitely high risk of bias. Q: question.

All studies included in the systematic review sought to present the protocols used to reproduce the in vitro gastrointestinal digestion of proteins of animal or vegetable origin, as only in vitro studies were found in the search, and none of the in-animal studies that were analyzed met the inclusion criteria. Therefore, only the OHAT tool was used to assess the risk of bias. The results presented (Table 3) follow the order according to the score obtained with the application of OHAT (Figure 2).

## 4. Discussion

Different enzymes can participate in the hydrolysis of protein molecules to obtain peptides, in isolation or combination, as occurs during the digestive process. Protease selection and hydrolysis duration are decisive factors in the types of peptides to be generated [51]. Furthermore, the type of enzyme, the temperature, the enzyme–substrate ratio, and the hydrolysis duration affect the degree of hydrolysis and, consequently, the generated peptides [52].

Notably, the stomach plays a critical role in the early stages of the digestion process. It is possible to highlight the role of specific cells in the gastric lining, known as chief cells, which release pepsinogen, requiring activation (pepsin) in an acidic environment. Therefore, pepsin is most effective in the pH range of 1.5 to 2, allowing cleavage of pepsinogen to form pepsin [53].

Considering that the stomach provides pepsin with an ideal environment for protein digestion, in vitro protocols are essential to ensure control of this environment and, based on reliable results, will provide a basis for experimental studies to be carried out with animals. In addition, we emphasize that in vitro methods are based on human physiology but are more straightforward, economical, and reproducible [54].

We highlight that none of the analyzed animal studies met the inclusion criteria, and thus, only in vitro studies were included in this review. The primary outcomes of the studies can be seen below. We emphasize that the resulting peptide/amino acid after in vitro digestion was not included in the inclusion criteria. This review aimed to gather experimental protein digestion protocols and not to obtain information and/or analyze the digestion products.

In this review, it was observed that 60% of the studies used protein sources of animal origin. Concerning the duration of the digestive phases, there was greater variation between studies for the oral phase. Most protocols used the enzymes amylase, pepsin, and pancreatin, with pH levels of 7, 3, and 7, respectively, for the oral, gastric, and intestinal phases. Thus, it is possible to perceive that different peptides can be released by simulated digestion since both the hydrolysis duration and the concentration of enzymes can influence the release and concentration of these peptides [51]. Furthermore, the different gastrointestinal compartments’ pH substantially impacts protein digestion since foods are exposed to variable pH conditions in the gastrointestinal tract, significantly influencing protein denaturation and local enzymatic activity [55].

According to Xavier and Mariutti [56], who carried out a review of the static and semi-dynamic in vitro digestion protocols currently applied to assess the bioavailability of different compounds, the effect of the digestion factors (enzymes, pH, incubation duration) between the other models is remarkable, a perception that corroborates the results of this review. Some protocols presented here probably needed to be appropriately described by the authors since important information about temperature and enzymes used were suppressed [21,23,24,26,28,29,30,34,35,38,40,42,45,46,47,48,50].

Regarding the protocols used, 13 studies adopted the INFOGEST protocol, which is suitable for studying protein digestion due to its similarity to human digestion [57]. Among the studies in this review, it was found that four carried out the in vitro simulation of the infant gastrointestinal tract, in which the same authors developed two. These researchers also simulated the digestion of the adult intestinal tract in the fed and fasting phases. Notably, care was taken in using different protocols for each life cycle and each state (fed or fasting), and in controlling the physiological conditions, because they present differences in stomach pH, enzymatic activity, and concentration of bile acids [24], and some proteins that escape digestion in the small intestine, for example, can be hydrolyzed by bacteria in the human colon [58].

In this context, the literature shows that numerous static and dynamic models of the in vitro simulation of the infant gastrointestinal tract have already been applied by researchers. Ménard et al. [25] compared in vitro gastric digestion in the infant model with that in the adult model and observed that, after 60 min of in vitro infant gastric digestion, about 62% of the proteins remained intact. Only 7% of the proteins remained unchanged in the adult model after gastric digestion. However, there is still a need to develop a harmonized static protocol for in vitro infant gastrointestinal digestion. With this, the main challenge is the precise definition of the consumer being created because digestive parameters are affected by age and clinical conditions, for example [59]. Therefore, future research should focus on methods to accurately simulate other age groups, such as infants, preschoolers, adolescents, the elderly, and even groups with specific disorders that affect normal digestion conditions [56].

It is essential to highlight that the standardized INFOGEST 2.0 in vitro digestion method, published in 2019, was improved, presenting recommendations for the oral phase, for example [6]. Despite being fast, we emphasize that the oral stage plays an essential role in the general digestive process and the rate of gastric emptying of solid foods [60]. Additionally, this improved version of the protocol helps to understand key steps in the process and preferred ways of stopping the digestion, depending on the study’s objective. Interestingly, among the articles that adopted the INFOGEST protocol in their research, seven were published after 2019 and did not adopt this updated version [19,22,24,28,29,30,47].

The protocols ranged from simple static models to highly sophisticated, computer-controlled dynamic gastrointestinal models. In static models, digestive fluid and food samples are constant. Therefore, they are convenient for investigating mass transport and structural breakdown mechanisms [6,20,37]. On the other hand, dynamic models include factors such as gastric emptying and an influx of gastric and intestinal juices [61,62]. It is worth noting that in dynamic models in the gastric phase, during the gradual reduction in pH, proteins can undergo aggregation and gelation, significantly impacting the digestion kinetics and protein profile being emptied from the stomach. In this context, dynamic models are physiologically more accurate, as the mentioned phenomenon is not observed in the static model [63,64].

However, only one study used dynamic digestion [36], and a second study employed the semi-dynamic model [23], which is based on a computer-controlled system but with sample proportions and enzyme activity similar to the recommendations of the INFOGEST in vitro static model. Another study adopted both static and dynamic digestion [30]. According to Brodkorb et al. [6], although accurate, dynamic models are inaccessible to most researchers because they require higher configuration and maintenance costs. This information may explain the small number of studies that adopted these models to perform gastrointestinal digestion.

Seven studies were performed in triplicate [21,24,26,29,30,42,47], and three in duplicate [34,45,50]. The others (*n* = 10) did not have this information reported. According to Passari et al. [65], there is an improvement in accuracy when studies are performed in triplicate instead of in duplicate, and performing in quadruplicate makes this precision excellent. Therefore, repetitions during the in vitro digestion process are essential to make studies more reliable. Another critical point to be explored concerns control groups, which provide confidence that the analytical method works well or detect cases in which an analytical method does not generate accurate results. In this review, all included studies had at least one control, i.e., the blank, which is used to highlight possible interferences capable of overlapping or masking the analyte signal [66].

Studies that adopted protein gastrointestinal digestion protocols, for the most part (*n* = 19), did not mention whether there was success in mimicking human digestion processes, probably because the main objective was to obtain peptides to perform other complementary analyses, such as of potential antioxidants, chemopreventive properties, and bioavailability, among others. Thus, considering that in vitro studies will serve as support for future investigations in animal studies, it is essential to emphasize that the technique used to perform gastrointestinal digestion, whether in human or animal studies, must be very precise, and capable of generating the same peptides in both processes, bearing in mind that in vitro conditions must be controlled based on the physiological conditions of the human gastrointestinal tract. Therefore, we suggest that future studies mention this ability to mimic the human protein digestion process so that analyses become more accurate and similar to what happens in humans.

Researchers compared protein gastrointestinal digestion based on the INFOGEST protocol with human or animal studies in this scenario. In the research by Sanchón et al. [34], which was included in this review, jejunal digestion investigated after oral ingestion of casein and whey protein was presented in vitro and in humans to study the identity of numerous peptide sequences, suggesting that the INFOGEST protocol approaches the final stages of the digestion gastrointestinal tract of milk proteins in humans. Egger et al. [57] discovered that there was a good correlation between protein degradation and the products generated at the end of the gastric phase with gastric peptides in an animal study using skimmed milk powder and compared the in vitro results with stomach samples and various sites of the intestine of pigs. Furthermore, there was a good correlation between the intestinal phase performed in vitro and samples from the jejunum of the animal study.

Another study carried out by Bohn et al. [67], with skim milk powder, compared the in vitro protein digestion of the INFOGEST protocol with the protein digestion in pigs, and it was noticed that the phases (gastric and intestinal) reflect the situation of the animals, considering protein digestion. The need for further research on in vitro–in vivo correlations with well-defined systems is evident so that the results are applied to analyze the bioavailability and digestibility of foods [68].

Another study, carried out by Egger et al. [69], also with skimmed milk powder, aimed to compare the standardized INFOGEST in vitro digestion protocol with the DIDGI^®^ dynamic protocol, and both with the protein digestion of pigs. It was observed that the parameters related to contents from the stomach and intestine of the two in vitro models were equivalent to those related to protein digestion in animal studies. Moreover, in the dynamic digestion protocol, the kinetics of protein hydrolysis, especially the release of free amino acids during the gastric phase, showed dynamic evolution and approached protein digestion in animal studies due to the constant addition of active enzymes.

Overall, the INFOGEST static protocol can be considered excellent for simulating the human protein gastrointestinal process since it has good laboratory reproducibility, power, ease of use, and greater accessibility, and enables a comprehensive evaluation of each stage of digestion, favoring its application for screening and studies involving mechanisms of action and formulation of hypotheses [6]. The main advantage of this protocol is the adequate standardization of experimental conditions and techniques [70]. However, it is known that there are limitations, and it is impossible to mimic the protein digestion dynamic process or the physiological interaction with the host using gastrointestinal digestion static models [6].

The intestinal phase is presented only as one stage, rather than sequential stages such as the duodenal, jejunal, and ileal phases, which present different dilutions, characteristics, pH levels, enzymatic actions, and microorganism contents. Thus, these limitations make it impossible to use this method to evaluate the detailed kinetics of the several stages of digestion [6]. However, considering the good correlation at the end of each phase of protein digestion between humans and animal models [34,57], the static model should only be used to evaluate these endpoints of digestion.

In human and animal protein cleavage studies, there are inter- and intra-individual variations, such as the enzyme–substrate ratio, enzyme secretion, and transit duration, which can affect digestibility endpoints. Considering the complexity, the enzymatic activity applied in in vitro assays can affect the reaction rate obtained and the final extent of substrate hydrolysis. In this context, a kinetic approach can contribute to a better understanding of the digestive action of several nutrients present in food during digestion [54].

The macro- and microstructure of foods can also change dynamically throughout gastrointestinal digestion, significantly affecting the kinetics of nutrient hydrolysis. Therefore, static and dynamic models have the role of clarifying different aspects of digestion. The static method is easy to apply to gain mechanistic insights into enzyme–substrate interactions and for screening samples for differences in digestibility. The dynamic process attempts to include physiologically relevant conditions and dynamic parameters to provide kinetic conditions of nutrient degradation patterns and structural changes in foods [54].

However, choosing the most accurate model of the human digestive process is complex. Selecting the most appropriate model requires careful evaluation of the study objectives to consider the advantages and limitations offered by each system. A compromise between technical complexity and physiological importance is often necessary to obtain reliable results [71].

Recently, an internationally standardized semi-dynamic in vitro digestion protocol was published by INFOGEST [72], which addresses the structural modifications of foods during the digestion process not considered in previous versions of static digestion [56], incorporating relevant dynamic parameters linked to the gastric phase (gradual acidification, and secretion of fluids and enzymes, in addition to emptying). It may be appropriate for several applications, combining the advantages of static and dynamic approaches [54].

Several studies show that indirect factors such as heating and high-pressure (HP) processing can affect rates and patterns of proteolysis. In this scenario, some authors observed that protein hydrolysis could generate more unfolded or flexible peptides, making it possible to use limited proteolysis as a processing method to increase the emulsifying properties of proteins. Although protein emulsification alters the structure of proteins, these molecules adsorbed by an oil droplet may also play a role in lipid digestion in the droplet. Given this, the in-depth knowledge can be used to design new colloidal foods, enabling food manufacturers and nutritionists to address substantial health issues such as obesity and cardiovascular disease [73].

Two main classes of physiological surfactants secreted in the gastrointestinal tract can significantly influence the kinetics of protein digestion: bile acids and phospholipids. In general, these have been shown to protect some types of protein from extensive degradation due to reduced flexibility. They have been shown to destabilize the structure of some proteins, making them more susceptible to proteolysis. In addition, bile acids are also able to play a role in the structural behavior of protein-based colloidal foods during duodenal proteolysis [73]. According to the study by Macierzanka et al. [74], carried out on the in vitro proteolysis of milk emulsion, bile acids introduced into the system during the duodenal phase of digestion are able to re-emulsify the oil that was separated due to gastric destabilization of the emulsion caused by pepsin or phosphatidylcholine.

The development of physiologically relevant in vitro digestion models has contributed significantly to knowledge about food structures in protein digestion. In addition, depending on the type and number of proteolytic enzymes, the digestion conditions, and the analysis of the protein hydrolysates used in in vitro digestion, there may be different digestibility results. Proteolytic digestion using three enzymes (trypsin, chymotrypsin, and peptidase) provides greater digestibility. In comparison, digestion with two enzymes (pepsin and pancreatin) provides greater reliability in distinguishing between samples of different digestibility because it occurs in two stages (gastric and intestinal) [75].

A study by Somaratne et al. [76] to investigate the role of biochemical digestion in the kinetics of softening and disintegration of egg white gels during the in vitro digestive process observed that softening and disintegration modes resulting from diffusion of gastric juice and mechanical tensions are dependent on the internal cohesive forces of the egg white gel matrices, which are related to their characteristics, such as texture and microstructure.

Floury et al. [77] verified microstructural changes in dairy gels of identical composition, differing only in the coagulation mode during in vitro gastrointestinal digestion, and concluded that there is a tendency for compact protein aggregates in rennet gels under acidic stomach conditions, which was not visualized with acidic gels. The kinetics of proteolysis were slower for the rennet gel, certainly due to the reduced accessibility of pepsin to its substrate.

A study by Ménard et al. [25] aimed to compare the digestive kinetics of commercial infant formula by applying two different in vitro gastric digestion models (infant model and adult model). The authors observed another behavior regarding pepsinolysis among the more resistant whey proteins with caseins, which were more extensively hydrolyzed. It is essential to highlight that, in vivo, caseins are digested more slowly than whey proteins. This occurs due to gelation in the stomach following the combined action of acid and pepsin. Manuscripts published in the literature that use semi- dynamic and dynamic models, as opposed to static models, demonstrate this fact. Based on this, the choice of model can significantly affect the results [78].

This can be justified by the structure of proteins, wherein caseins have a loose and flexible structure, making them more susceptible to pepsinolysis. In contrast, serum proteins have a globular and compact structure [79]. Furthermore, it is important to highlight that whey proteins and caseins are digested differently in different consumers, such as babies, adults, and the elderly, due to the organism’s physiology [80,81,82].

According to Shani-Levi et al. [59], several studies have sought to rationally process, structure, and formulate foods to maintain health and promote the well-being of consumers. Thus, it is possible to highlight the modern food production system’s efforts to develop food distribution systems that protect bioactive ingredients. In this way, there will be controlled release in the gastrointestinal tract, with consequent modulation of the colon microbiome or induction of satiety, for example. However, it is necessary to understand the principles that guide the digestive fate of food. Thus, we emphasize that this understanding can become clearer through in vitro gastrointestinal digestion studies.

In addition, it is worth mentioning that the health benefit generated by dietary proteins is achieved after hydrolysis by gastrointestinal digestion since this biological process can influence the peptide profile of foods, either by degrading bioactive peptides or releasing new peptides [83]. Thus, considering the resistance of some peptides to digestion and knowing that others can be released during displacement through the gastrointestinal tract, the digestive process greatly impacts the generation of the peptides [52]. Therefore, it is reinforced that the protocols used to simulate human protein digestion should reflect with more accuracy the human physiological conditions.

It is important to highlight that many approaches have been applied to generate in vitro models of the human digestive system [84]. Considering the methodological protocol used, this review presented a discussion based especially on static, semi-dynamic, and dynamic models.

However, there are still organoids, which are structures obtained from the in vitro cell culture of human embryonic stem cells (ESCs) or induced pluripotent stem cells (iPSCs) derived from already differentiated adult tissues, healthy or not, that generate three-dimensional biological structures (3D), in vitro [85]. In this context, digestive tissues have also been obtained through this cell culture, and its use has proven interesting for understanding the mechanisms of various diseases, including the development of medicines [86]. It would be the same for understanding the digestive process of proteins. Organoids are known to recapitulate many biological characteristics of human organs, including tissue heterogeneity, the characteristics of the spatial distribution of different cells, cell–cell and cell–matrix interactions, and some functions that arise from specific cells of the tissue obtained [87,88,89]. Thus, they are more representative of in vivo physiology [90], so they fill gaps in the in vitro models discussed here. Therefore, they may be promising for providing a deeper understanding of the functionality of the digestive organs, and have great potential for recapitulating the complex pathogenesis that involves this system, thus accelerating the applications of personalized medicine [86].

Furthermore, working with 3D cellular structures undoubtedly allows for a better approximation of what occurs in the studied organ. However, even with all these advantages, these structures cannot be recognized as “mini-organs”. The technique used to obtain organoids does not generate a miniature of a complete functional or vascularized organ; it only generates a tissue part of it. Several complex tissues (including the liver, which is essential to the digestive process) are made up of different cell types and pose a challenge in reproducibility from organoids, which do not yet have a heterogeneous proportion of cells that reflect the tissue studied. Furthermore, even if it were possible to reproduce an entire organ in the laboratory, there would be no guarantee that it would function identically to what happens in the human body.

Finally, although some in vitro protein digestion systems reported in the literature can be statistically correlated with protein digestion processes in human and animal studies, it is essential to consider the anatomy and morphology of the upper gastrointestinal tract in the development of more advanced and biologically relevant in vitro protein digestion systems, given their important role in determining the rate and extent of digestion [71].

## 5. Conclusions

Dynamic, static, and even semi-dynamic models can all assist in reproducing gastrointestinal protein processes. However, the static model can be highlighted as the most accessible and, therefore, the most used. Human and animal studies are used to compare and ensure the greater precision and reliability of the results of in vitro experiments. A comparison of the static model used in most studies (70%) with human or animal studies showed that this protocol is adequate for studying the human gastrointestinal digestion of proteins due to its similarity with digestion in humans. Therefore, we conclude that static models can simulate human proteolytic digestion, and its limitations are minimized, if the physiological conditions are considered and controlled.

This experimental research is fundamental to providing specific and reliable scientific information capable of generating mechanistic insights that mimic human hydrolysis to generate peptides. Thus, the information obtained in this review can be a helpful foundation in in silico and human or animal studies, as it will help in the choice of strategies for processes and studies involving enzymatic hydrolysis. Because no animal studies were found that met the inclusion criteria defined in this review, we recommend that these studies be carried out with greater methodological rigor. Thus, combining several in vitro gastrointestinal protein digestion protocols can allow a faster and more appropriate method to be followed, depending on the study’s objective.

## Figures and Tables

**Table 1:** Search strategies used for each database.

Database	Search Strategies
PubMed and Web of Science ^1^	digestion AND gastrointestinal tract AND protein hydrolysate AND (“in vitro” OR “rat” OR “mice” OR “animal”)
Science direct ^2^	digestion AND gastrointestinal tract AND “digestion process” (“protein hydrolysate” OR peptide) AND “in vitro” AND animal
EMBASE and Biblioteca Virtual em Saúde	digestion OR digestibility AND gastric AND (peptides OR proteins) AND (“in vitro” OR animal)
Scopus ^1^	“digestion process” AND “gastrointestinal tract” AND (peptides OR proteins) AND (“in vitro” OR mice)

^1^ In the search in the Web of Science and Scopus databases, the filter *Document type (article*). ^2^ In the search in the Science Direct database, the filter *Research articles*. EMBASE: Excerpta Medica Database.

**Table 2:** Eligibility criteria (inclusion and exclusion) of studies following PICOS ^a^.

PICOS	Inclusion	Exclusion
**Population**	Original articles resulting from in vivo studies performed with rats and mice of both sexes and different ages (puppies, young, adults, or elderly) without water or diet restriction, cell studies, and in vitro (gastrointestinal simulating fluids).	Articles with digestion models with other animals, or studies with computer simulations (in silico)
**Interventions**	Studies in which the intervention group has been submitted to the administration of peptides, proteins, or gastrointestinal simulating fluids to reproduce digestive processes of peptides and/or proteins	Studies that mimic the digestion process with the application of non-protein molecules or these molecules associated with peptides and/or proteins
**Controls**	Studies that present the control group composed of animals, cells, or in vitro experiments without administration of peptides or proteins.	Studies without a control group
**Outcomes**	Studies describing the model used to mimic protein digestion.	Studies that do not describe the protocol to simulate gastrointestinal conditions or studies that do not present schedule, experiment duration, frequency, administered dosages, concentration, and temperature, or studies that do not mimic digestion in humans
**Study design**	In vitro and in vivo experimental studies	Other types of studies
**Other (article type)**	Published original research articles	Other types of studies

^a^ PICOS: Population, interventions, controls, outcomes, and study design.

**Table 3:** Study characteristics selected for the systematic review.

Reference	Samples Used	Enzymes Used	pH	Temperature (Experimental/Interruption)	Experiment Duration (Each Step)	Protocol Adopted ^1^
[19]	Defatted Cashew Flour (DCF)	α-amylase, pepsin, and porcine pancreatin	7; 3 and 7	37 °C/90 °C	Oral phase: 10 min. Gastric phase: 120 min. Intestinal phase: 120 min.	[20]
[21]	Beef, chicken, pork, and turkey	Porcine α-amylase, pepsin, pancreatin	2 (pepsin); the others were not reported	37 °C/Not reported	Oral phase: 5 min., Gastric phase: 120 min., Intestinal phase: 120 min.	INFOGEST [20]
[22]	Z. *bungeanum* seed protein	Pepsin, trypsin solution	3 and 7	37 °C/60 °C	Gastric phase: 120 min., Intestinal phase: 120 min.	[20]
[23]	Human milk (HM) or infant formula (IF)	α-amylase, lipase, porcine pepsin, porcine pancreatin	6.3 (salivary fluid with amylase); 5.8 (gastric fluid with lipase and pepsin); 7.0 (intestinal fluid)	37 °C/Not reported	Gastric phase: 120 min., Intestinal phase: 180 min.	Semi-dynamic in vitro simulation of the infant gastrointestinal tract
[24]	Peanut Ara h 1; wheat gliadin for bread; bread or peanuts	α-amylase from human saliva (for solid samples: peanuts and bread), porcine pepsin, porcine trypsin, bovine chymotrypsin, pancreatin from porcine pancreas (solid samples).	7 (oral phase—infant and adult fasting); 5.3 (gastric phase); 6.6 (intestinal phase)—infant/3 (gastric phase); 7 (intestinal phase)—fed adult; 1.2 (gastric phase), 7 (intestinal phase)—fasting adult	37 °C/Not reported	Oral phase (for solid samples: peanuts and bread): 2 min., Gastric phase (infant digestion and fed adult-static): 60 min., Intestinal phase (infant digestion and fed adult-static): 60 min.	The oral phase for the three protocols (infant, adult fed, and adult fasting) was based on the INFOGEST protocol. The gastric and intestinal phases for the infant protocol were based on Ménard et al. [25]. Static in vitro digestion for fed adults was based on the INFOGEST protocol
[26]	Kefir	α-amylase and mucin, pepsin, pancreatin	2–3 (gastric), 7 (intestinal)	37 °C/Not reported	Oral phase: 5 min., Gastric phase: 120 min., Intestinal phase: 120 min.	[27]
[28]	Three isolated proteins (zein, whey protein, and collagen) and five foods (peanuts, sorghum flour, wheat bran cereals, pigeon peas, and black beans)	Amylase, pepsin, pancreatin	7; 3 and 7	37 °C/Not reported	Oral phase: 2 min., Gastric phase: 120 min., Intestinal phase: 120 min.	INFOGEST [20]—Static digestion
[29]	Bovine milk proteins β-lactoglobulin (BLG) and β-casein (BCS), whole fresh cow’s milk, and large free-range hard-boiled eggs	Pepsin from porcine gastric mucosa, trypsin from porcine pancreas and bovine chymotrypsin (intestinal digestion of isolated proteins), pancreatin from the porcine pancreas for intestinal meal phase and infant and adult fed models	5.3—gastric and 6.6—intestinal (infant); 3 and 7 (adult fed); 1.2 and 7 (fasting adult)	37 °C/Not reported	Gastric phase: 60 min., Intestinal phase: 60 min.	[25] (infant) and INFOGEST—[20] (adults)
[30]	Pea Protein Concentrate (PPC) 5% or 15% (static and dynamic)	α-amylase, pepsin and pancreatin (static)/amylase, porcine pancreatic lipase, porcine gastric mucosal pepsin and pancreatin (dynamics)	7; 3 and 7 (static)/No reported (dynamics)	37 °C/freezing with liquid nitrogen (static), 37 °C/Not reported (dynamics)	Oral phase: 2 min. Gastric phase: 120 min. Intestinal phase: 120 min. (static)/Total digestion time: 240 min. (dynamics)	INFOGEST [20]—Static digestion + Minekus [31]; Denis et al. [32]; Ribnicky et al. [33]—Dynamic digestion
[34]	Casein and whey protein powders	Pepsin from porcine gastric mucosa, pancreatin from porcine pancreas	No reported	37 °C/Quick freezing with liquid nitrogen	Gastric phase: 20 and 120 min., Intestinal phase: 60 min. and 120 min.	[20]
[35]	Whey Protein Isolate (WPI)	Fresh stock solution of porcine pepsin, pancreatin	3 and 7	37 °C/Not reported	Gastric phase: 120 min., Intestinal phase: 120 min.	INFOGEST [20]
[36]	Milk protein dissolved in water	Pepsin	1.5	37 °C/90 °C	Total digestion time: 220 min.	Simulated gastric fluid was prepared according to [20], and dynamic digestion was performed according to Kong and Singh [37]
[38]	Soy and whey protein hydrolysates	Pepsin, lipase, pancreatin	4.39 (stomach) and 6.8 (intestinal)	37 °C/Not reported	Gastric phase: 40 min., Intestinal phase: 120 min.	Nguyen et al. [39] (infant digestion)
[40]	Milk supplemented with Brewers’ spent grain (BSG)	Pepsin, pancreatin	2 and 7.4	37 °C/Not reported	Gastric phase: 60 min., Intestinal phase: 120 min.	McCarthy et al. [41]
[42]	Cranberry juice with whey protein isolate	Pepsin, pancreatin	7 (spittle), 2–3 (stomach) and 7 (intestine)	37 °C/Not reported	Oral phase: 30 s, Gastric phase: 120 min., Intestinal phase: 120 min.	Bornhorst and Singh [43] and Roman et al. [44]
[45]	Quinoa Protein Concentrate (QPC)	Porcine pepsin, porcine pancreatin	No reported (gastric)/7 (intestinal)	37 °C/Quick freezing with liquid nitrogen	Gastric phase: 120 min., Intestinal phase: 120 min.	[20]
[46]	*Bresaola* ground	α-amylase, pepsin, pancreatin	7; 3 and 7	Not reported	Oral phase: 2 min., Gastric phase: 120 min., Intestinal phase: 120 min.	INFOGEST [20] Static digestion
[47]	Casein Aggregates (CAs) and Egg White Proteins (EWPs)	Porcine pepsin	4	37 °C/Not reported	Gastric phase: 120 min.	INFOGEST [20]—Static digestion
[48]	Cod and chicken protein hydrolysates	No reported	No reported	Not reported	No reported	Tibäck et al. [49] (static digestion)
[50]	Kiwicha Protein Concentrate (KPC)	Porcine pepsin, porcine pancreatin	No reported	Not reported	Gastric phase: 120 min., Intestinal phase: 120 min.	[20]

^1^ In vitro studies.

## Data Availability

All data generated or analyzed during this study are included in this article. Further inquiries can be directed to the corresponding author.

## Supplementary Materials

The following supporting information can be downloaded at: https://www.mdpi.com/article/10.3390/nu16152398/s1, Table S1: Office of Health Assessment and Translation (OHAT) questions; File S1: Preferred Reporting Items for Systematic Review and Meta-Analysis Protocols (PRISMA-P) checklist; File S2: Protocol registered in the International Prospective Register of Systematic Reviews (PROSPERO). Reference [91] is cited in the supplementary materials.
