# Supplementary material for: Experimental Protocols Used to Mimic Gastrointestinal Protein Digestion: A Systematic Review
Source: Nutrients. 2024 Jul 24;16(15):2398. doi: 10.3390/nu16152398 (PMC11314321; doi:10.3390/nu16152398)
Supplement: Supplementary file 1 [file nutrients-16-02398-s001.zip › nutrients-2984995-supplementary.pdf]

# What are the digestion and absorption models used to reproduce gastrointestinal protein processes? a protocol for systematic review

## Citation

Rafael Costa, Anna Luz, Gidyenne Medeiros, Grasiela Piuvezam, Ana Morais. What are the digestion and absorption models used to reproduce gastrointestinal protein processes? a protocol for systematic review. PROSPERO 2020 CRD42020198709 Available from: https://www.crd.york.ac.uk/prospero/display_record.php?ID=CRD42020198709

## Review question [2 changes]

- What are the animal, cell and / or in vitro models of protein digestion that mimic gastrointestinal processes in humans?

- What are the animal, cell and / or in vitro models of protein absorption that mimic intestinal processes in humans?

So the research question is:

- P: Rats or mice, human or animal cells, gastrointestinal simulating fluids.

- I: Administration of proteins or methodology with simulating fluids to perform the gastrointestinal process.

- C: Protein digestion and absorption studies (intervention group) / Protein-free studies (control group).

- O: Gastrointestinal digestion and absorption protocol of the test protein / peptide

Primary outcome: description of the protocol

Secondary outcome: gastrointestinal digestion and intestinal absorption of the test protein / peptide

- S: Animal, cell and / or in vitro studies (gastrointestinal simulating fluids)

## Context and rationale

Recently, with the advancement of new technologies, the emergence of bioactive molecules that are candidates for clinical application in the treatment of obesity has been growing steadily. Several researches presenting models with cells, animals and in vitro have shown to be excellent alternatives. In this sense, numerous models are available to mimic the gastrointestinal tract and, thus, show how these biomolecules, especially proteins, behave when exposed to this scenario / environment. Thus, this protocol will have two systematic reviews as a product, in the first one it is expected to identify the protocols used in cell, animal and / or in vitro models, related to the digestive processes of proteins and / or peptides; in the second, it is intended to focus on protocols with cells, animals and / or in vitro related to the absorptive processes of proteins and / or peptides. From the studies in question, which will have the content and methodological quality critically evaluated, it will be possible to assist the scientific community in the treatment of obesity and its comorbidities (type 2 diabetes, systemic arterial hypertension, changes in the lipid profile and inflammatory markers, through the clinical application of peptides and bioactive proteins, enabling the understanding of the products of protein digestion and intestinal permeability, in addition to the stability of these proteins during the stages of digestion and absorption. To this end, it is necessary to understand the gastrointestinal processes during the administration of proteins, since the gastrointestinal environment can affect the functionality of these proteins.

## Searches [2 changes]

Searches will be conducted in electronic bibliographic databases: PubMed; ScienceDirect; Scopus; Web of Science; Evidence Portal, of the Virtual Health Library; EMBASE.

The search equation for the systematic review regarding gastrointestinal digestion and intestinal absorption will be defined considering the following items: In vivo or cell or in vitro studies (gastrointestinal simulating fluids), intervention (oral administration of proteins or application of process simulating fluids gastrointestinal), results (gastrointestinal digestion models or intestinal absorption models), results study design (intestinal absorption of the protein / peptide component).

## Study designs to be included [2 changes]

### Inclusion criteria:

Experimental studies in vivo or in vitro (gastrointestinal simulating fluids) that assess digestion or protein absorption will be included.

### Exclusion criteria:

Case studies, cross-studies, studies without a separate control group and studies in silico will be excluded.

## Human disease modelled [1 change]

Obesity and comorbidities

## Animals/population [1 change]

### Inclusion criteria:

Rats or mice of both sexes and ages (puppies, young, adults or elderly), without water or diet restrictions and in vitro studies (gastrointestinal simulating fluids) and cells

### Exclusion criteria:

Experimental models with other animals or population and in silico studies.

## Intervention(s), exposure(s) [2 changes]

### Inclusion criteria:

Studies with peptides or proteins as treatment. All studies involving schedule, experiment time, frequency, dosages administered, concentration and temperature are eligible for inclusion.

### Exclusion criteria:

Studies that do not describe the protocol used, schedules, time of experience, frequency, doses administered to animals, cells or in vitro (gastrointestinal simulating fluids) or that do not present sufficient data regarding the dosage or

characteristics of the sample, as well as studies that do not mimic gastrointestinal processes.

## Comparator(s)/control [3 changes]

### Inclusion criteria:

In vivo, cell and in vitro studies (gastrointestinal simulator fluids) that evaluate the use of peptides or proteins and mimic gastrointestinal processes.

### Exclusion criteria:

Studies without a control group

## Other selection criteria or limitations applied [2 changes]

Only scientific publications with original data will be included. Review studies and gray literature will not be included. There will be no time or language limitations.

## Outcome measure(s) [1 change]

### Inclusion criteria:

Studies that describe the model used to mimic protein digestion or absorption.

### Exclusion criteria:

Studies that do not describe the protocol used to simulate gastrointestinal conditions by applying peptides or proteins.

## Study selection and data extraction [3 changes]

### Procedure for study selection

The selection of articles will be carried out by reading the title of the article and abstract at the first moment. In the second moment, there will be a complete reading of the selected articles. In both moments, the articles will be read independently by two researchers. Discrepancies will be resolved by a third reviewer.

### Prioritise the exclusion criteria

Title-abstract screening:

1. Articles that do not have protocols to simulate gastrointestinal conditions.

2. Articles with models of digestion and absorption with other animals or studies in silico.

3. Studies with other molecules of non-protein origin.

4. Works that do not specify schedules, time of experience, frequency, doses administered and concentration.

5. Studies that do not specify information, such as the progress of the experiment, frequency of doses administered.

6. Studies without at least one control group.

7. Documents that are not scientific articles.

### Methods for data extraction

Two reviewers will independently extract data from each article. A third reviewer will resolve discrepancies. A spreadsheet will be created and the data of interest for each review will be inserted (numerical data, graphs or tables). If any data is not detailed in the article, we will contact the authors to request detailed information.

### Data to be extracted: study design

Gastrointestinal digestion models, intestinal absorption models, control groups and components that are part of the digestive or absorption process.

### Data to be extracted: animal model

The data to be extracted will include:

- Articles with animals - animal species, animal lineage, life stage, sex, protocol adopted to simulate gastrointestinal processes.

- Article with cells - Origin of the cell, genetic background of the cells (normal or cancerous), types of cell line (intestinal epithelium).

- In vitro studies - Composition of gastrointestinal simulator fluids, type of substance used, enzymes used, time and temperature of gastrointestinal processes.

### Data to be extracted: intervention of interest

Dose, timing of administration, frequency of administration, route of administration, vehicle.

### Data to be extracted: primary outcome(s)

Detailed description of the model used to simulate digestion and absorption of proteins in animals, cells or in vitro (simulating fluids), including in animal studies the process of group randomization, accommodation, method used to simulate digestion and absorption in animals. For cell and in vitro work, the exposure time, temperature, digestion phases, gastric volume, enzyme concentration and activity, pH, volume of the gastrointestinal simulator fluids, chemical reagents used and type of test protein / peptide will be extracted. In vitro studies will also remove data on the conditions related to the composition of the gastrointestinal simulator fluids and the conditioning of the samples after all stages of digestion (stored at 80ºC or lyophilized).

### Data to be extracted: secondary outcome(s)

Fragmented peptides (categorical data) and absorbed fragmented peptides (categorical data).

### Data to be extracted: other

First author, year of publication and newspaper.

## Risk of bias and/or quality assessment [1 change]

By use of SYRCLE’s risk of bias tool. Other criteria, namely OHTA (Office of Health Assessment and Translation).

For animal studies, the risk of study bias will be assessed using SYRCLE's Risk of Bias (RoB) instrument. This tool is specifically designed to assess the risk of bias in animal studies (HOOIJMANS et al, 2014). The OHAT tool (Services, 2019) to assess the risk of bias will be used to assess the risk of bias in cell and in vitro studies..

## Strategy for data synthesis

### Planned approach

The data will be summarized by a narrative approach and the characteristics of the included studies will be described in tables.

The studies related to the reviews will be structured around the phases of digestion and / or absorption, considering the experimental conditions, including volume of the simulating fluids, types of substances used in each phase of digestion and / or absorption, culture conditions, type of protein simulation test, pH, time and temperature, as well as techniques and tools for studying in vitro and vivo digestion and absorption.

For both reviews, summaries of the results of the different studies will be provided, as well as the similarity of the protocols in mimicking digestion and / or absorption in vivo. The data will be presented in summary tables and in narrative forms to describe the characteristics of the included studies. These data will be structured with the type of protein used for simulation of digestion and absorption, animal species and lineage, dose, type of administration, treatment time and way of conducting the experiment.

### Effect measure

Not applicable

### Effect models

Not applicable

### Heterogeneity

Not applicable

### Other

Not applicable

## Analysis of subgroups or subsets [1 change]

### Subgroup analyses

No subgroup analysis is planned.

### Sensitivity

Not applicable.

### Publication bias

Not applicable.

## Contact details for further information

Rafael Costa

rafaeloliveira.nutri@gmail.com

## Organisational affiliation of the review

Universidade Federal do Rio Grande do Norte (UFRN)

https://sigaa.ufrn.br/sigaa/public/programa/portal.jsf?id=1639

## Review team members and their organisational affiliations

Mr Rafael Costa. Universidade Federal do Rio Grande do Norte (UFRN)

Ms Anna Luz. UFRN

Ms Gidyenne Medeiros. UFRN

Grasiela Piuvezam. UFRN

Ana Morais. UFRN

## Review type [1 change]

Pre-clinical animal intervention review

## Anticipated or actual start date [2 changes]

15 December 2020

## Anticipated completion date [2 changes]

20 September 2021

## Funding sources/sponsors [1 change]

Coordenação de Aperfeiçoamento de Pessoal de Nível Superior (CAPES)

## Conflicts of interest

## Language

English

## Country

Brazil

## Stage of review

Review Ongoing

## Subject index terms status

Subject indexing assigned by CRD

## Subject index terms

Animals; Gastrointestinal Tract; Physical Phenomena; Physiological Phenomena; Protein Processing, Post-Translational

## Date of registration in PROSPERO

25 August 2020

## Date of first submission

14 July 2020

## Details of any existing review of the same topic by the same authors

Give details of earlier versions of the systematic review if an update of an existing review is being registered, including full bibliographic reference if possible.

## Stage of review at time of this submission

The review has not started

| Stage | Started | Completed |
|---|---|---|
| Preliminary searches | No | No |
| Piloting of the study selection process | No | No |
| Formal screening of search results against eligibility criteria | No | No |
| Data extraction | No | No |
| Risk of bias (quality) assessment | No | No |
| Data analysis | No | No |

## Revision note

Dear After a new reading of the protocol, it was verified that some corrections would be necessary. Therefore, we request authorization to perform them. We clarify that the modifications signaled will not impact significant changes. However, they are required considering the question sent - What are the digestion and absorption models used to reproduce gastrointestinal protein processes? And the perception of adjustments to make the record more transparent, providing accurate information to reduce any problem involving the record and the respective complete creation of the systematic review.

*The record owner confirms that the information they have supplied for this submission is accurate and complete and they understand that deliberate provision of inaccurate information or omission of data may be construed as scientific misconduct.*

*The record owner confirms that they will update the status of the review when it is completed and will add publication details in due course.*

## Versions

25 August 2020

25 August 2020

26 March 2021

| Section/topic | # | Checklist item | Information reported | | Line number(s) |
|---|---|---|---|---|---|
| | | | Yes | No | |
| **ADMINISTRATIVE INFORMATION** | | | | | |
| **Title** | | | | | |
| Identification | 1a | Identify the report as a protocol of a systematic review | ☒ | ☐ | 1 |
| Update | 1b | If the protocol is for an update of a previous systematic review, identify as such | ☐ | ☒ | NA |
| **Registration** | 2 | If registered, provide the name of the registry (e.g., PROSPERO) and registration number in the Abstract | ☒ | ☐ | 48 |
| **Authors** | | | | | |
| Contact | 3a | Provide name, institutional affiliation, and e-mail address of all protocol authors; provide physical mailing address of corresponding author | ☒ | ☐ | 5 e 61 |
| Contributions | 3b | Describe contributions of protocol authors and identify the guarantor of the review | ☒ | ☐ | 275 |
| **Amendments** | 4 | If the protocol represents an amendment of a previously completed or published protocol, identify as such and list changes; otherwise, state plan for documenting important protocol amendments | ☐ | ☒ | NA |
| **Support** | | | | | |
| Sources | 5a | Indicate sources of financial or other support for the review | ☒ | ☐ | 266 |
| Sponsor | 5b | Provide name for the review funder and/or sponsor | ☒ | ☐ | 266 |
| Role of sponsor/funder | 5c | Describe roles of funder(s), sponsor(s), and/or institution(s), if any, in developing the protocol | ☐ | ☒ | NA |
| **INTRODUCTION** | | | | | |
| **Rationale** | 6 | Describe the rationale for the review in the context of what is already known | ☒ | ☐ | 65 |
| **Objectives** | 7 | Provide an explicit statement of the question(s) the review will address with reference to participants, interventions, comparators, and outcomes (PICO) | ☒ | ☐ | 101 |
| **METHODS** | | | | | |
| **Eligibility criteria** | 8 | Specify the study characteristics (e.g., PICO, study design, setting, time frame) and report characteristics (e.g., years considered, language, publication status) to be used as criteria for eligibility for the review | ☒ | ☐ | 116 |
| **Information sources** | 9 | Describe all intended information sources (e.g., electronic databases, contact with study authors, trial registers, or other grey literature sources) with planned dates of coverage | ☒ | ☐ | 158 |
| **Search strategy** | 10 | Present draft of search strategy to be used for at least one electronic database, including planned limits, such that it could be repeated | ☒ | ☐ | 159 |
| ***STUDY RECORDS*** | | | | | |
| Data management | 11a | Describe the mechanism(s) that will be used to manage records and data throughout the review | ☒ | ☐ | 170 |
| Selection process | 11b | State the process that will be used for selecting studies (e.g., two independent reviewers) through each phase of the review (i.e., screening, eligibility, and inclusion in meta-analysis) | ☒ | ☐ | 184 |
| Data collection process | 11c | Describe planned method of extracting data from reports (e.g., piloting forms, done independently, in duplicate), any processes for obtaining and confirming data from investigators | ☒ | ☐ | 185 |
| **Data items** | 12 | List and define all variables for which data will be sought (e.g., PICO items, funding sources), any pre-planned data assumptions and simplifications | ☒ | ☐ | 188 |

| | | | | | |
|---|---|---|---|---|---|
| **Outcomes and prioritization** | 13 | List and define all outcomes for which data will be sought, including prioritization of main and additional outcomes, with rationale | ☒ | ☐ | 188 |
| **Risk of bias in individual studies** | 14 | Describe anticipated methods for assessing risk of bias of individual studies, including whether this will be done at the outcome or study level, or both; state how this information will be used in data synthesis | ☒ | ☐ | 200 |
| ***DATA*** | | | | | |
| **Synthesis** | 15a | Describe criteria under which study data will be quantitatively synthesized | ☐ | ☒ | NA |
| | 15b | If data are appropriate for quantitative synthesis, describe planned summary measures, methods of handling data, and methods of combining data from studies, including any planned exploration of consistency (e.g., $I^2$, Kendall's tau) | ☐ | ☒ | NA |
| | 15c | Describe any proposed additional analyses (e.g., sensitivity or subgroup analyses, meta-regression) | ☐ | ☒ | |
| | 15d | If quantitative synthesis is not appropriate, describe the type of summary planned | ☒ | ☐ | 209 |
| **Meta-bias(es)** | 16 | Specify any planned assessment of meta-bias(es) (e.g., publication bias across studies, selective reporting within studies) | ☐ | ☒ | NA |
| **Confidence in cumulative evidence** | 17 | Describe how the strength of the body of evidence will be assessed (e.g., GRADE) | ☐ | ☒ | NA |

From: Moher D, Liberati A, Tetzlaff J, Altman DG, The PRISMA Group (2015). Preferred reporting items for systematic review and meta-analysis protocols (PRISMA-P) 2015 Statement. Systematic Reviews, 2015, 4(1):1-9. doi: 10.1186/2046-4053-4-1.

BioMed Central
The Open Access Publisher

| | Question |
|---|---|
| 1 | Was administered dose or exposure level adequately randomized? |
| 2 | Was allocation to study groups adequately concealed? |
| 3 | Did selection of study participants result in appropriate comparison groups? |
| 4 | Did the study design or analysis account for important confounding and modifying variables? |
| 5 | Were experimental conditions identical across study groups? |
| 6 | Were the research personnel and human subjects blinded to the study group during the study? |
| 7 | Were outcome data complete without attrition or exclusion from analysis? |
| 8 | Can we be confident in the exposure characterization? |
| 9 | Can we be confident in the outcome assessment? |
| 10 | Were all measured outcomes reported? |
| 11 | Were there no other potential threats to internal validity (e.g., statistical methods were appropriate and researchers adhered to the study protocol)? |
